# Highly efficient enzymatic biodiesel production promoted by particle-induced emulsification

**DOI:** 10.1186/s13068-015-0247-6

**Published:** 2015-04-03

**Authors:** Juan Mangas-Sánchez, Patrick Adlercreutz

**Affiliations:** Department of Biotechnology, Lund University, P.O. Box 124, Lund, SE-221 00 Sweden

**Keywords:** Biodiesel, Lipase, Transesterification, *Thermomyces lanuginosus*, High fatty acid content, Ethanolysis, Pickering emulsion and two-phase system

## Abstract

**Background:**

At present, the conversion of oils to biodiesel is predominantly carried out using chemical catalysts. However, the corresponding lipase-catalysed process has important advantages, which include mild reaction conditions and the possibility of using cheap, low quality feedstocks with a high free fatty acid content. Further increases in the efficiency of the enzymatic process are desired to make it even more attractive and suitable for large-scale applications.

**Results:**

Herein, we present a simple and efficient two-phase lipase-catalysed system for the preparation of biodiesel in which different parameters (biocatalyst composition, ethanol concentration and the presence of additives) were optimised in order to obtain the maximum productivity starting from triolein with a high free oleic acid content. In the two-phase system, the enzyme tolerated high-ethanol concentrations, which made it possible to reach high conversions. The addition of silica particles increased the reaction rate substantially. It was suggested that such particles can catalyse acyl migration as a step to the full conversion to glycerol and biodiesel. However, in the system studied here, the effect of the particles was shown to be due to the formation of smaller and more uniform emulsion droplets leading to better mass transfer between the two phases. Particles of widely different size had positive effects, and the highest rate was obtained with silica particles derivatised with phenyl groups. The optimal conditions were applied to the solvent-free ethanolysis of rapeseed oil, and a yield of 96% was reached in 5 h. Under the mild conditions used, chemical catalysts were inefficient.

**Conclusions:**

Triacylglycerol oils with a high free fatty acid content can be efficiently converted to ethyl esters using *Thermomyces lanuginosus* lipase as the catalyst in an aqueous/organic two-phase system. Fast mass transfer can be achieved using silica particles, which helped to decrease the size of the emulsion droplets and thus led to a more efficient process. The high-ethanol concentration tolerated by the lipase in this system made it possible to reach almost quantitative yields.

## Background

Biodiesel is a mixture of esters that can be produced by the transesterification of triglycerides (oils) with short-chain alcohols, obtaining glycerol as the major by-product [1-3]. Biodiesel production has gained importance in recent years for its ability to replace fossil fuels since it can be blended with conventional diesel fuel. Additionally, its use does not require any important technical modifications on the engine, and it provides environmental benefits since it is a renewable source of energy [4]. Global biodiesel production was less than 1 billion litres in 2000, which has increased to almost 15 billion litres in 2009 [5]. The EU is the world’s main producer, with a market share of around 55%. This production is highly influenced by domestic fuel policy [6], and in this context, several targets have been established for the use of biodiesel in fuels [7].

Industrial biodiesel production is still mainly carried out via base-catalysed transesterification using sodium or potassium hydroxides with an excess of methanol [8-11]. Alkoxides are also often used since less water is released in the reaction. These processes present several drawbacks such as the need to remove inorganic salt in the downstream process, the high temperature required and undesirable side reactions. Also, these systems have been found to be inefficient when a high free fatty acid (FFA) content is present in the starting material [12], which restricts the use of conventional chemical pathways to a highly pure feedstock. For ecological and economical reasons, the development of new methodologies has attracted attention in recent years. In this context, the use of lipases may become an alternative solution [13-16]. Immobilised lipase-catalysed transesterification in the presence of an organic solvent has been studied in detail. The possibility of reusing the enzyme and the simplicity of the downstream process makes this approach attractive [17]. While the use of biocatalytic methods is well-established for the preparation of pharmaceutical products, much higher productivities are required for commodity chemicals due to the price of biocatalysts. Concerning biodiesel production, lipase immobilisation adds an extra cost to the process and partial inactivation may also occur. Therefore, the catalyst has to be reused many times in order to be economically feasible [18]. For these reasons, the use of liquid lipase preparations has attracted interest, but the optimisation of these processes is needed in order to establish them as a realistic industrial alternative.

Two-phase systems are of particular interest for reactions with both non-polar and polar substrates and products. Lipases specifically evolved for the hydrolysis of fats and oils; they are activated in biphasic mixtures in a process called interfacial activation [19]. Most lipases have a lid covering the lipophilic active site. Once the lipase comes into contact with a non-polar surface, the lid opens, exposing the lipophilic area where the active site is situated. This part is oriented towards the organic layer, such that the lipid can easily enter the active site and the hydrophilic products can diffuse into the aqueous layer. For that reason, two-phase reaction media seem to be the most suitable environment for these particular enzymes [20]. In this sense, we have recently developed an efficient methodology for the chemoenzymatic preparation of 2-monoolein and 1,2-diolein in a two-phase system [21]. Nevertheless, these systems present mass transfer limitation issues, especially when working with surfactant-like species such as diacyl and monoacylglycerides (DAGs and MAGs). To overcome this limitation, the use of strategies which can favour mass transfer in the complex system is important.

In the current study, aqueous-organic two-phase systems for biodiesel production were developed. In the development work, a substrate containing triolein rich in free oleic acid was used to simulate a low-quality oil. Since the substrate contained no fatty acids other than oleic acid, it was possible to quantify all intermediate acylglycerol products accurately, thus facilitating the optimization of the system in a rational way. Finally, the best reaction conditions were also applied to the solvent-free conversion of rapeseed oil to biodiesel.

## Results and discussion

### Enzyme screening

**Figure 1:**
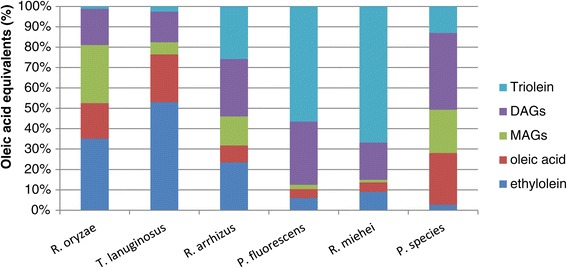
**Product composition after 6 h using different lipases.** Ethanolysis of triolein catalysed by different lipases at 37°C and 1,000 r.p.m using 400 mM ethanol and 75 mg enzyme/g substrate for solid preparations and 100 μL enzyme/g substrate for liquid formulations. DAGs, diacylglycerols; MAGs, monoacylglycerols.

Enzymatic ethanolysis of triglycerides (TAGs) for the preparation of biodiesel constitutes a complex system because it includes two liquid phases and several enzyme-catalysed reactions. Lipases express different specificities for each reaction involved [16], and therefore, the screening of lipases in the reaction system is important. Initially, both the organic and aqueous phase were analysed, but it was found that the amounts of acylglycerols, biodiesel and FFA in the aqueous phase were negligible. Lipases from *Rhizomucor miehei* and *Pseudomonas fluorescens* converted only small amounts of TAG under the conditions used (Figure 1). The lipase from *Pseudomonas* sp. converted most of the TAG, but only minor amounts of biodiesel were formed; instead, DAG, MAG and FFA were the main products. *Rhizopus oryzae*, *Rhizopus arrhizus* and *Thermomyces lanuginosus* lipases produced biodiesel yields >23%. *R. oryzae* and *R. arrhizus* lipases express strong 1,3-specificity and promoted the accumulation of high 2-MAG concentrations. *T. lanuginosus* lipase was efficient in converting all the acylglycerols and was found to be the most suitable biocatalyst for biodiesel preparation. Using this lipase, 97.5% conversion of TAG was reached after 6 h, providing a 53% yield of biodiesel.

### Biocatalyst composition

**Figure 2:**
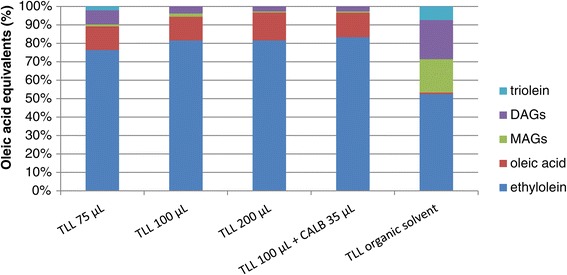
**Product composition after 24 h using different enzyme amounts and combinations.** Ethanolysis of triolein catalysed by different lipase types and amounts at 37°C, 1,000 r.p.m. and 400 mM ethanol concentration. DAGs, diacylglycerols; MAGs, monoacylglycerols; TLL, *T. lanuginosus* lipase.

The biodiesel yield improved with an increasing amount of TLL up to 100 μL of enzyme per gram of substrate (Figure 2). It has been shown that combinations of lipases can favour the process since different lipases have different substrate specificity [16,22]. As a complement to TLL, which is efficient in converting TAG, *Candida antarctica* lipase B (CAL-B) was chosen since this enzyme can efficiently convert DAG and MAG [16]. However, under the conditions used, the addition of CAL-B did not improve the biodiesel yield. It should be pointed out that the observed yields are higher than those obtained using immobilised TLL in a low-water system with a similar substrate (Figure 2, [16]), which shows that TLL is more efficient in biodiesel production in a two-phase system than in a pure organic medium. This agrees with previous observations that TLL is very efficient in aqueous/organic two-phase systems rather than in pure organic media compared to CAL-B [23]. However, a drawback of the two-phase system is that the high-water content causes a significant hydrolysis reaction, which competes with biodiesel formation.

### Effect of ethanol concentration

**Figure 3:**
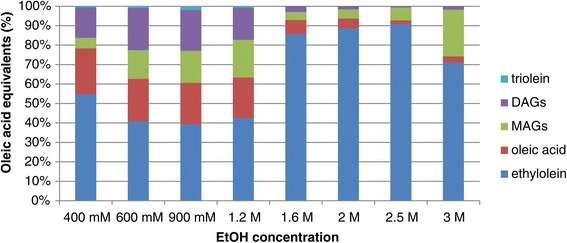
**Product composition after 6 h using different ethanol concentrations.** Ethanolysis of triolein catalysed by TLL lipase using different ethanol concentrations and 100 μL of TLL/g substrate at 37°C and 1,000 r.p.m. DAGs, diacylglycerols; MAGs, monoacylglycerols.

In order to suppress the hydrolysis reaction and favour the alcoholysis reaction, the alcohol concentration in the reaction mixture can be increased, although it is known that alcohols can also inhibit and inactivate lipases [24,25]. A range of ethanol concentrations between 400 mM and 3 M was tested. The initial rate was highest at ethanol concentrations of 1.6 and 2.0 M (0.17 μmol min^−1^ μL^−1^ enzyme), while after 6 h, the highest biodiesel yield was observed with 2.5 M ethanol (Figure 3). After 24 h, equilibrium was reached in most cases, and the yield increased with an increasing ethanol concentration, with the exception of the highest one (Table 1). It should be noted that the FFA concentrations decreased towards the end of the reaction, which indicates that the FFA formed under kinetic control was esterified in the later part of the reaction, when equilibrium was approached. An additional experiment was conducted using the same experimental set-up with 100 mM oleic acid and 2 M ethanol as the substrates. After just 1 h, 95% conversion was observed, showing that the enzyme was able to convert FFA into biodiesel efficiently. It seemed that the enzyme tolerated high-ethanol concentrations in the two-phase system quite well and a concentration of 2 M was chosen for the rest of the experiments.

**Table 1:** **Product composition using different amounts of ethanol**

	**400 mM**	**600 mM**	**900 mM**	**1.2 M**	**1.6 M**	**2 M**	**2.5 M**	**3 M**
Ethylolein	72.1 ± 2.4	73.3 ± 3.1	78.5 ± 2.2	91.0 ± 2.7	98.3 ± 1.4	98.0 ± 1.6	99.7 ± 0.2	99.4 ± 0.3
Oleic acid	24.6 ± 2.2	15.5 ± 0.7	11.7 ± 2.1	5.9 ± 3.6	1.7 ± 1.4	2.0 ± 1.6	0.3 ± 0.2	0.6 ± 0.3
MAGs	0.8 ± 0.2	3.5 ± 0.7	4.7 ± 0.7	3.1 ± 0.6	0	0	0	0
DAGs	2.5 ± 0.8	7.7 ± 0.6	5.1 ± 0.1	0	0	0	0	0
Triolein	0	0	0	0	0	0	0	0

Product composition (oleic acid equivalents, %) after 24 h at 37°C and 1,000 r.p.m. using different ethanol concentrations and 100 μL of TLL/g substrate. DAGs, diacylglycerols; MAGs, monoacylglycerols.

### Effects of added particles

**Figure 4:**
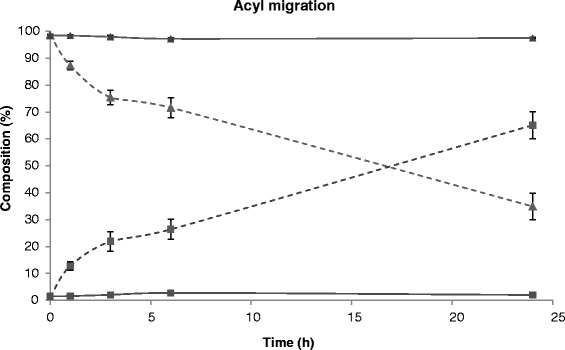
**Acyl migration study.** The 2-monoolein (triangles) and 1-monoolein (squares) as a function of time using 4% *w*/*v* silica gel (35- to 70-μm particle size) as acyl migration promoter in the two-phase system (*n*-heptane/water 20%, solid lines) and in pure *n*-heptane (dotted lines).

**Figure 5:**
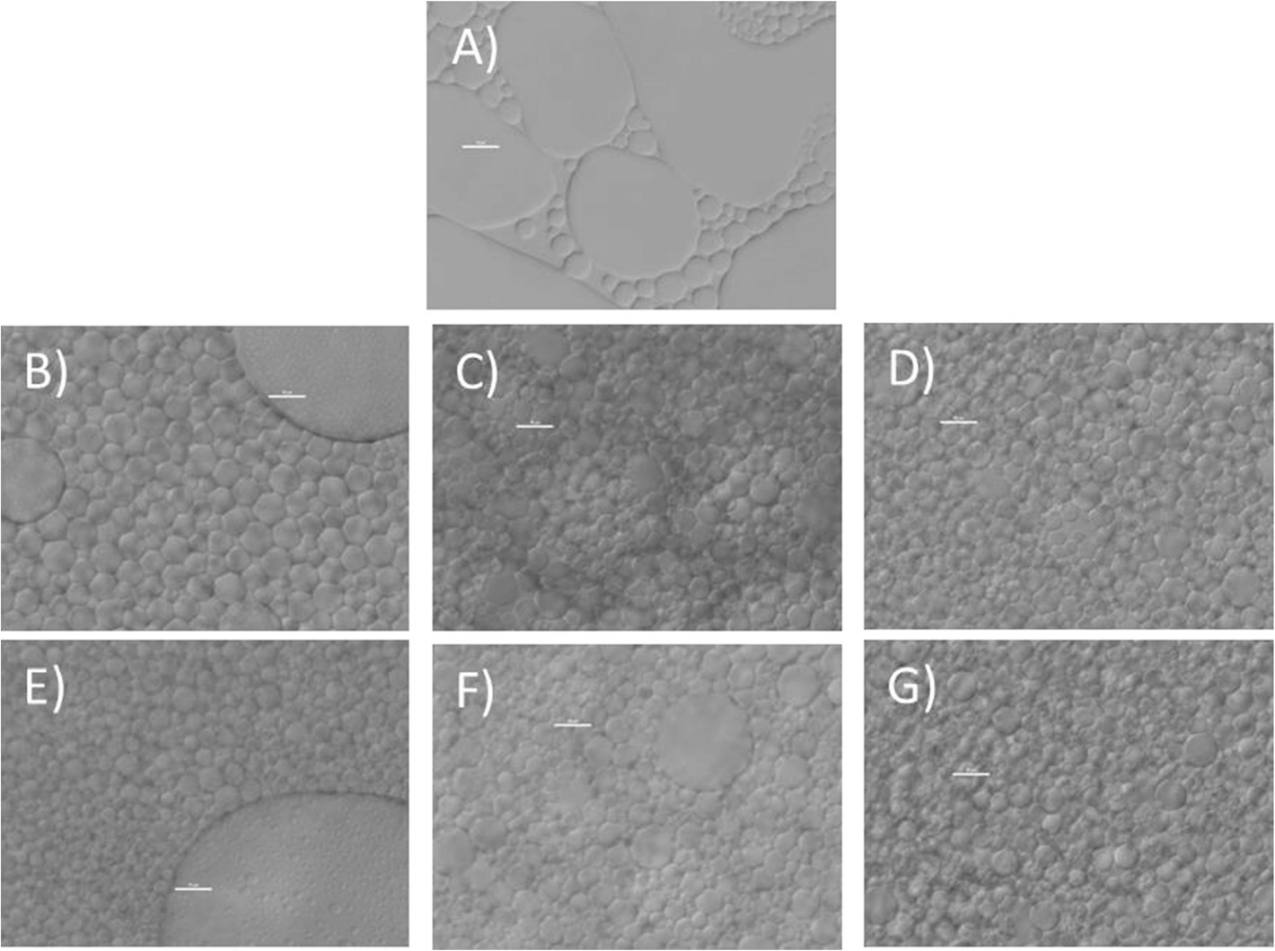
**Emulsion pictures using different conditions. (A)** No additive, **(B)** non-modified silica gel 35 to 70 μm, **(C)** non-modified silica gel 0.2 to 0.3 μm, **(D)** non-modified silica gel 0.007 μm, **(E)** phenyl silica gel 35 to 70 μm, **(F)** phenyl silica gel 0.2 to 0.3 μm, **(G)** phenyl silica gel 0.007 μm. Used was 4% *w*/*v* of the different additives and 100 μL of TLL/g substrate. Scale bars: 10 μm. The large structures in (B) and (E) are silica particles.

Due to the high 1,3-specificity expressed by many lipases, including TLL, the accumulation of 2-MAG is expected to constitute a bottleneck in the process. To promote the overall conversion to glycerol and biodiesel, the addition of substances able to catalyse the isomerisation of 2-MAG to the thermodynamically more stable 1(3)-MAG, which is a better substrate for lipases, has been applied successfully. In a typical example, soybean oil is converted to biodiesel using a mixture of immobilised lipase and silica gel as the catalyst [26]. Another approach is to immobilise the lipase on an anion exchange resin, which works both as a support for the lipase and as an acyl migration catalyst [27]. Since silica is the most commonly used acyl migration catalyst, the addition of 4% *w*/*v* silica (35- to 70-μm particle size) was evaluated in the reaction system used in the present study; it was found to increase the rate of biodiesel formation significantly. In order to confirm that it was acyl migration that caused the increased conversion rate, 2-monoolein was synthesised separately and its isomerisation to 1(3)-monoolein was studied under different conditions. Acyl migration was promoted by silica in pure heptane, in agreement with previous observations (Figure 4). However, surprisingly enough, no acyl migration was observed in the water/heptane two-phase system in the presence or absence of silica (Figure 4). In order to determine the mechanism of the silica-induced increase in the biodiesel production rate, the reaction mixtures were subjected to microscopic investigation. It was found that the presence of silica particles caused the formation of an emulsion with smaller and more uniform droplets than in the absence of silica particles (Figure 5). It is likely that the increased interfacial area caused the observed increase in the reaction rate, by speeding up the mass transfer of substrates and products between the phases. Alternatively, the increased rate could have been due to increased interfacial activation of TLL due to the increased interfacial area.

**Figure 6:**
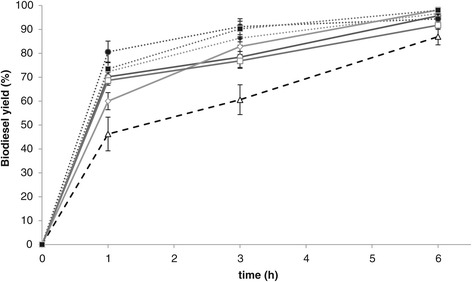
**Biodiesel batch production (yield) using different solid particles.** No additive (dashed line), phenyl-modified silicas (dotted lines, closed symbols) and non-modified silicas (solid lines, open symbols). Particle size: 35 to 70 μm (circles), 0.2 to 0.3 μm (diamonds) and 0.007 μm (squares). Used was 4% *w*/*v* of the different additives and 100 μL of TLL/g substrate.

**Figure 7:**
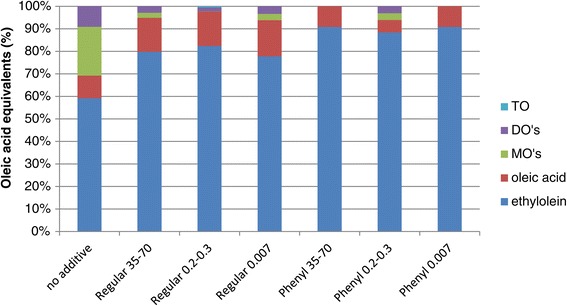
**Product composition after 3 h using different silica gels.** Ethanolysis of triolein catalysed by TLL using regular silica or phenyl silica with different particle size (given in microns) as additives and 100 μL of TLL/g substrate at 37°C and 1,000 r.p.m. DO, dioleins; MO, monoolein; TO, triolein.

It is known that small solid particles can act as amphiphilic molecules, adsorbing on droplets and preventing flocculation and coalescence and thus creating so-called Pickering emulsions [28-32]. However, this requires much smaller particles and more power input than the shaking that was used in the present study. To further study the effects of particles on the studied biodiesel process, the addition of smaller silica particles was evaluated. Furthermore, since it has been shown that not only the size but also the surface character of the particles can affect the stability of the emulsion [33], all sizes of silica particles were evaluated in a phenyl-derivatised form as well. All types of particles increased the biodiesel formation rate and the phenyl-modified particles were somewhat more efficient (Figure 6). After a 3-h reaction time, all acylglycerols had been converted in the reactions involving Phenyl 35–70 and Phenyl 0.007 (Figure 7), and in the continued reaction, a further increase in yield was observed due to esterification of the originally formed FFA. After 6 h, yields over 95% were obtained regardless of the particle size and hydrophobic character.

All particles caused the formation of emulsions with a similar droplet size (Figure 5). Since the effect was observed not only with nanoparticles, but also with particles much larger than the liquid droplets, it is clear that this is not a typical example of a Pickering emulsion. Furthermore, the power input was most probably not high enough to create a Pickering emulsion, which normally requires ultrasound, high-pressure homogenizers or other devices producing high-shear stress [31]. However, our emulsions induced by particles without the use of high-energy emulsification equipment may be quite useful for biodiesel production because of the considerably increased reaction rate. The fact that slightly higher rate enhancement was observed with phenyl-derivatised silica particles indicates that an additional activation mechanism might have been in operation. It is possible that the lipase to some extent was adsorbed on the hydrophobic surface of the derivatised particles, thereby causing opening of the lid and interfacial activation, as previously observed after adsorption of lipases on hydrophobic materials. However, since almost equal activation was observed with underivatised silica particles, it appears that the main rate-enhancing effect of the particles was due to the reduction of droplet size.

### Comparison with chemical catalysis

Methanolysis of vegetable oils in the presence of a homogeneous base catalyst is nowadays the dominant process in industrial biodiesel production. A comparison between the biocatalytic method developed in this work and the homogeneous catalyst under the same conditions (pressure, temperature and time) was carried out. The most common acid/base homogeneous catalysts used in industry, for example, NaOH, NaOMe, KOH and H_2_SO_4_ were included in the comparison at a concentration of 2% (*w*/*v* or *v*/*v*). No reaction was detected in any case after 24 h. These results can be explained by the high free fatty acid content of the starting material and also by the mild reaction conditions used.

### Biodiesel preparation from rapeseed oil

Once the optimised conditions were set, these were applied to a real feedstock material. Rapeseed oil was selected as the substrate, and the reaction was carried out under solvent-free conditions, starting from 300 mg of oil, 0.4 mL of water, 8 mg of phenyl silica (35- to 70-μm particle size) and 100 μL of enzyme/g substrate at 37°C and 1,000 r.p.m. The process was monitored by GC, and after 5 h, 96% biodiesel yield was observed, with 2% MAGs and 2% DAGs remaining.

## Conclusions

We have developed a very effective method for the enzymatic preparation of biodiesel, which is also useful for oils with a high free fatty acid content. The method uses a liquid formulation of *T. lanuginosus* lipase in an aqueous/organic two-phase system. The enzyme has shown an excellent tolerance to ethanol, which not only made the process faster but also allowed for the total conversion of the free fatty acid into biodiesel with a short-reaction time. Triacylglycerol conversion was very fast, while the main bottleneck in the process was the conversion of DAG and MAG. The use of solid silica particles led to changes in the emulsion structure which permitted a larger surface area in the interphase, overcoming mass transfer limitations and thus increasing the rate of the process. Surprisingly, the silica particles did not cause acyl migration as observed in other biodiesel production systems. The emulsion-enhancing effects of particles of widely varying size has not been described before, at least not in the biodiesel production field, and is clearly different from the creation of Pickering emulsions, which requires small particles and high-energy input. This work demonstrates the potential of liquid lipase preparations for biodiesel production starting from high free fatty acid feedstocks such as waste oils. Under the best conditions, almost quantitative yields were achieved after only 6 h of reaction. These results constitute a good starting point for efficient and cheap biodiesel production, and the next step would be to adapt the methodology to readily available cheap waste oils.

## Materials and methods

### Chemicals

Lipases from *T. lanuginosus* (2,200 U/mL, Sigma), *R. oryzae* (Aldrich, product number 80612), *R. arrhizus* (Sigma, product number 62305), *P. fluorescens* (Aldrich), *R. miehei* (Sigma), *Pseudomonas species* (Aldrich) and *Candida antarctica* lipase B (Sigma), *p*-nitrophenyl butyrate and silica gels of different particle sizes (silica gel 35 to 79 μm, silica fumed 0.2 to 0.3 μm and silica fumed 0.007 μm) were obtained from Sigma-Aldrich Co. (Steinheim, Germany); cyclohexane and tetradecane were purchased from Merck (Darmstadt, Germany); *n*-heptane, ethanol (99.5%) from VWR (Stockholm, Sweden); and *N*-methyl-*N*-trimethylsilylheptafluorobutyramide (MSHFBA, Machery-Nagel) from ScantecLab (Partille, Sweden). Triolein was obtained from Larodan Fine Chemicals (Malmö, Sweden), perfluorodecyltriethoxysilane from Alfa Aesar and phenyltrimethoxysilane and *n*-octyltriethoxysilane were acquired from Acros Organics. Rapeseed oil was purchased in a local supermarket. Other chemicals were of analytical grade. A high oleic acid content starting material was used in order to simulate a low-quality oil. The composition (molar ratio) of the starting material was 54% triolein, 24% DAGs and 22% oleic acid.

### GC analysis

The samples were analysed by GC using a Varian gas chromatograph (430-GC-FID, Agilent Technologies Inc., Santa Clara, CA) equipped with a flame-ionisation detector and a FactorFour™ capillary column (VF = 1 ms, length 15 m and ID 250 μm, Varian, Agilent Technologies Inc., Santa Clara, CA). Helium was used as the carrier gas, and the temperature of both the detector and injector was 350°C. The starting temperature of the column was 180°C. This was maintained for 2.5 min, then increased by 10°C/min to a temperature of 340°C and was then maintained for 26 min.

Samples were withdrawn from the organic phase and derivatised by silylation by adding an equal volume of MSHFBA and incubating the mixture at room temperature for 30 min. An equal volume of dry ethanol was then added to stop the reaction. The samples were diluted 30 times with an internal standard solution (tetradecane) in cyclohexane to obtain a sample in which the final solution of tetradecane was 9 mM, suitable for GC. The concentrations of the compounds were calculated using response factors obtained from a standard curve.

### Alcoholysis reactions

The standard reaction conditions were 265 mg of the starting material (mainly triolein, see the section on ‘Chemicals’) in 1.6 mL of *n*-heptane with different ethanol concentrations in a 4.5-mL vial with septum. The reaction was started by adding the biocatalyst (different amounts) in 0.4 mL of H_2_O. The reactions were carried out in a thermomixer at 1,000 r.p.m. Samples from the organic phase were regularly withdrawn with a Hamilton syringe and analysed by GC. The concentration of each component was calculated as a percentage of the initially available oleic acid equivalents, bearing in mind that diacylglycerols and triacylglycerols contain two and three fatty acid moieties, respectively. All experiments were carried out in duplicate. Error bars are omitted in some charts in order to have a better understanding of the results since standard deviations are in all cases below 4%.

### Silica modification

Silica gels of different particle sizes were modified with phenyl moieties in order to increase their hydrophobicity. The most often used method for preparing hydrophobic inorganic support materials is to silanise the support. To that purpose, 200 mg of silica (30- to 75-, 0.2- to 0.3- and 0.007-μm particle sizes) were suspended in 1.5 mL of dry toluene, and the mixture was kept at 130°C for 6 h. Then, 0.5 mL of phenylmethoxysilane was added, and the mixture was stirred overnight at 70°C. The suspension was cooled and the solvent was removed by decantation. The preparation was washed with fresh toluene, and MTBE and was finally air-dried at room temperature.

### Microscopy

The structure of the emulsions was studied by interference microscopy. Images were taken using a Nikon Optishot-2 microscope at × 400 magnification with Digital Sight DS 2Mv camera and processed using NIS-elements 3.1D.

## Abbreviations

CAL-B: *Candida antarctica* lipase B
DAGs: diacylglycerols
GC: gas chromatography
MAGs: monoacylglycerols
MSHFBA: *N*-methyl-*N*-trimethylsilylheptafluorobutyramide
MTBE: methyl *tert*-butyl methyl ether
TLL: *Thermomyces lanuginosus* lipase
